# Sequence Dynamics of Pre-mRNA G-Quadruplexes in Plants

**DOI:** 10.3389/fpls.2019.00812

**Published:** 2019-06-27

**Authors:** Piotr M. Kopec, Wojciech M. Karlowski

**Affiliations:** Department of Computational Biology, Faculty of Biology, Mickiewicz University in Poznań, Poznań, Poland

**Keywords:** G-quadruplex, RNA, sequence variability, plants, Arabidopsis, rice

## Abstract

Intramolecular G-quadruplexes (G4s) are secondary structures that may form within G-rich stretches of nucleic acids. Although their presence has been associated with genomic instability and mutagenicity, recent reports suggest their involvement in regulation of diverse cellular events, including transcription and translation. The majority of data regarding G4s stems from mammalian and yeast studies, leaving the plant G4s almost unexplored. Using the publicly available *Arabidopsis thaliana* and *Oryza sativa* WGS data, we examined the single nucleotide variability of sequences predicted to form G4s (pG4s) structures. We focused our analysis on protein coding transcripts and compared the results to well-characterized *Homo sapiens* data. We demonstrate that the overall high variability of pG4s is not uniform and differs between gene structural elements. Specifically, plant AUG-containing pG4s, located within 5′UTR/CDS junctions, are abundant and appear not to be affected by a higher frequency of sequence change, indicating their functional relevance. Furthermore, we show that substitutions lowering the probability of G4s’ formation are preferred over neutral or stabilizing modifications.

## Introduction

G-quadruplexes (G4s) are secondary structures of nucleic acids that can form within or with co-participation of guanine (G) rich strands. G4s’ core is a stack of planarly organized guanine tetrads, stabilized by centrally located cations (Dolinnaya et al., 2012; Rhodes and Lipps, 2015). Computational analyses revealed that the sequences predicted to form G-quadruplexes (pG4s) are not uniformly distributed along the human genome. pG4s seem to be preferentially localized within the proximity of transcription start sites (TSS) (Huppert and Balasubramanian, 2007), and untranslated regions (UTRs) (Huppert et al., 2008). This observation suggested that G4s might represent biologically relevant structures and a number of subsequent studies implicated their role in the regulation of various cellular processes. DNA G4s have been shown to be involved in regulation of transcription (Fedeles, 2017; Fukuhara et al., 2017) and telomere maintenance (Moye et al., 2015). RNA G4s participate in regulation of translation (Song et al., 2016; Lee et al., 2017), splicing (Huang et al., 2017; Weldon et al., 2018), alternative polyadenylation (Beaudoin and Perreault, 2013), miRNA binding (Rouleau et al., 2017), and telomere maintenance (Hou et al., 2017; Bao and Xu, 2018). Apart from their biological activity, stability and tunability of pG4s makes them a promising tool in nanobiotechnology (Lu et al., 2016; Tian et al., 2016; Lin et al., 2017).

Since the majority of available experimental data comes from mammalian and fungal systems, the role of G4 structures in plants’ physiology remains unclear. Genomic distribution of plant pG4s is not uniform, and varies between species (Mullen et al., 2010; Takahashi et al., 2012; Andorf et al., 2014; Wang et al., 2015; Garg et al., 2016; Griffin and Bass, 2018). Interestingly, there is a clear distinction between monocot and dicot plants in terms of pG4s density and localization – monocots show a significantly higher pG4s content (Wang et al., 2015; Garg et al., 2016; Griffin and Bass, 2018). An analysis of distribution of genic pG4s in *Oryza sativa* revealed their enrichment within 5′UTRs (Wang et al., 2015). In contrast, analogous analysis of *Arabidopsis thaliana* showed that the majority of genic pG4s is localized in the coding region (CDS) (Mullen et al., 2010; Wang et al., 2015). Experimental evidence for G4 function in plants remains rather sparse. Already reported examples include 5′UTR G4 acting as a repressor of translation (Kwok et al., 2015) and G4s located within tRNA-derived fragments (tRFs) acting as a mild modulator of translation efficiency *in vitro* (Jackowiak et al., 2017).

Although high-throughput experimental methods for G4s prediction are available (Chambers et al., 2015; Hänsel-Hertsch et al., 2018), computationally based analyses still remain a viable alternative for comparative studies on multiple genomes. A classical model of intramolecular G-quadruplex assumes that a single G-tetrad consists of guanines from four neighboring G-tracts, separated by loops. In accordance with this view, a number of G4 studies focused on sequences matching (G_n_N_1-k_)_3_G_n_ pattern (Huppert and Balasubramanian, 2005), where n restricts the number of G-tetrads and k confines the length of the loop. However, it has been shown that the classic definition of G4 might lead to their underestimation, because many G-rich sequences not following this pattern are still able to adopt G4 conformation (Chambers et al., 2015; Varizhuk et al., 2017). This issue has been addressed by authors of G4Hunter algorithm (Bedrat et al., 2016), which features a more general approach by abolishing pattern dependence. It additionally aims to reduce false-positive discovery rate by accounting for G/C skewness of the strand, which dictates competition between G4s and alternative structures.

Properties of G4s’ structure indicate that it can be easily compromised by a single point mutation (Chaudhary et al., 2017; Zeraati et al., 2017). Therefore, a mutation rate assessment of pG4s sequences may be a good indicator of their biological relevance, especially since G4s themselves are thought to be factors contributing to genomic instability (Lemmens et al., 2015). In this view, high evolutionary stability (low variability) indicates functional relevance and, in contrast, a high variability might suggest detrimental or neutral character of G4s. A previous study suggested that human pG4s are depleted of SNPs, especially at sites predicted to be most crucial for structural integrity (Nakken et al., 2009). A recent study based on high-throughput data showed depletion of disruptive mutations in pG4s in the vicinity of TSS. However, the general variant density was higher in pG4s than in the random sequences (Zhao et al., 2010). Large genomic studies, like 1001 Genomes Project (1001 Genomes Consortium, 2016) or 3000 Rice Genomes Project (3000 Rice Genomes Project, 2014), now offer an exciting opportunity to explore these properties of pG4s in plants.

In this study, we address the question of pG4s variability in plants. Specifically, we investigate whether the pG4s variant density differs from the background. Furthermore, we try to assess the biological significance of the variability, by examining the impact of the observed variants on the probability of G4 formation. We narrow down the pool of studied pG4s to those located in the coding strand of genes, and thus possibly forming in pre-mRNA after transcription. The single-stranded nature of RNA, its structure forming potential, and wide spectrum of phenotypic effects following mRNA processing alterations make RNA G4s especially interesting. We focus on the model dicot plant *A. thaliana* and a cultivated monocot, *O. sativa* ssp. japonica, using high-throughput data. The choice of plant models was dictated by the availability of a large amount of quality intraspecific data, contributed by the aforementioned studies. Additionally, we compare our results to a well-characterized reference model – *Homo sapiens.* This allowed us to treat ‘plants’ as a unit in reference to this distant mammalian model. Simultaneously, such a comparison provided an opportunity to contrast previous findings concerning human pG4 variability with our plant-based results. We show that pG4s are overall variable regions, which accumulate more variants potentially destabilizing the structures.

## Materials and Methods

### Genomic Sequences, Re-sequencing, Variant and Annotation Data

*A. thaliana* reference genome was downloaded from NCBI (GI: 332189094, 330250293, 332640072, 332656411, 332002898), corresponding annotation Araport11 v. June 2016 (Cheng et al., 2017) was downloaded from Araport database (Krishnakumar et al., 2015). Raw sequencing data of 1135 lines from 1001 Arabidopsis Genomes Project was obtained from SRA (project SRP056687). Human reference genome GRCh37 and 1000 genomes variant phase3 callset were acquired from 1000 genomes project ftp server (Auton et al., 2015). GRCh37.87 annotation was acquired from Ensembl. *O. sativa* Nipponbare reference genome MSU7 and corresponding annotation were retrieved from Rice Genome Annotation Project (Kawahara et al., 2013). 3000 Rice Genomes Project 29mio SNP callset against MSU7 was downloaded from Rice SNP-Seek Database (3000 Rice Genomes Project, 2014; Mansueto et al., 2017). All genomes were masked using RepeatMasker 4-0-7 (Smit et al., 2013–2015), with dfam2.0 (Hubley et al., 2016), RMBlast 2.6.0 and RepBaseRepeatMaskerEdition-20170127 data (Bao et al., 2015).

### Read Mapping and Variant Calling

Read mapping, and subsequent variant calling, was performed for *A. thaliana* only. Mapping of 1135 lines was conducted using BWA-mem (Li, 2013), with default options. Duplicate reads were removed with Picard^1^, unmapped and unpaired reads were filtered out with samtools (Li et al., 2009; Li, 2013). Variant calling was performed with GATK HaplotypeCaller, variants were then combined and hard-filtered following GATK’s Best Practices recommendations (Van der Auwera et al., 2013).

### pG4s Identification and Annotation

Potential G-quadruplexes were extracted from reference genomes using custom Python implementation of G4Hunter algorithm. Three identification runs were performed, with parameters: window size (ws) 20, threshold (t) 1.7; ws 30, t 1.4 and ws 25, t 1.6. Results were merged with mergeBed [bedtools package (Quinlan, 2014)] and resulting intervals were rescored with G4Hunter. Our goal was to maximize the input data, risking possibility of FDR elevation. However, in the case of the variant analysis in an evolutionary context, such not-perfect sequences were equally interesting. For further steps, we selected pG4s localized entirely on the coding strand of protein coding genes, and pG4s were annotated with overlapping gene’s IDs. We assigned pG4s to structural regions of the genes: 5′UTRs, 3′UTRs, Introns, and CDS (exons without UTRs). Additionally, we included 5′UTR/CDS, 3′UTR/CDS and Intron/Exon regions defined as fragments spanning from -30 to +30 from the actual feature junction. pG4s were assigned to a particular region only if they were entirely contained within it. All operations were performed using the bedtools suite.

### pG4s Prevalence and Distribution Analysis

The density of unique pG4s in every structural region was calculated in relation to merged intervals. To estimate the pG4 enrichment, we performed a simulation by randomly shuffling pG4s’ intervals along the genic coordinates and recalculating resulting pG4 densities in structural regions. After 300 iterations, we obtained theoretical random distributions of pG4 densities across the regions, which were subsequently used to calculate enrichment/depletion values and their respective *p*-values. pG4 enrichment value was then calculated for every region by dividing observed pG4 densities by mean values of simulated expected distributions of pG4 density.

### pG4s Variability Analysis

To assess the variability of pG4s in a given structural feature (e.g., 5′UTR), we calculated mean SNP densities of the feature and mean SNP densities of pG4s within the feature. The statistical significance of the difference between expected and observed counts of pG4s variants was determined by *X*^2^-test. To plot the pG4s variant density within the genomic context, we first determined a number of variants on every position of every pG4 and their 30 bp long flanking regions. Then, the part of the array corresponding to pG4s underwent linear interpolation to the arbitrary length of 30, using Interp1d function from scipy.interpolate package. The resulting set of arrays of the length of 90 was averaged and the result was plotted. The selection of pG4s, variant count of which significantly differed from their regions of localization, was conducted on the basis of a Fisher’s exact test.

## Results

### ΔG4Hscore Analysis

To determine whether there is a preference toward variants stabilizing or destabilizing pG4s, we first calculated the background probabilities of 12 possible substitutions for every genic region. Then, for every variant position within pG4s, we calculated the expected absolute change of G4Hscore (ΔG4Hscore), i.e., the average of 3 possible substitutions weighted by their background probabilities. In this way, we simulated a theoretical distribution of change of G4Hscore, which we compared with the distribution of the observed change. Next, to constrain the positional effect of substitutions, we simulated second distribution of ΔG4Hscore, this time modeling impact of only those positions that actually contain variants in our callsets. Their effect on ΔG4Hscore was weighted by their background substitution probabilities. The significance of the difference between observed and theoretical distributions of ΔG4Hscore was assessed using Wilcoxon rank sum test.

For all statistical tests we assumed α = 0.05. For all plots, we used ggplot2 R package (Wickham, 2016).

### Comparative Analysis of pG4s Distribution

**FIGURE 1:**
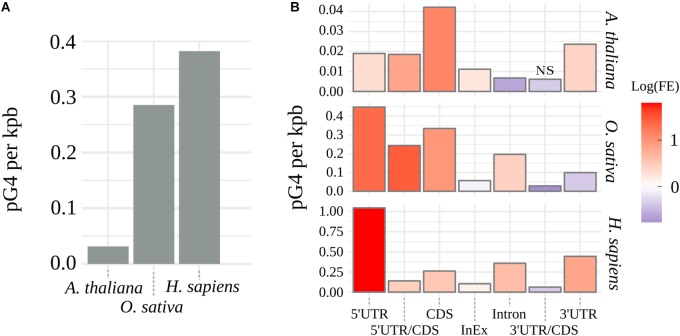
Distribution of pG4s. **(A)** Density of mRNA pG4s in thale cress, human and rice. **(B)** Enrichment of pG4s in structural elements of genes: red for overrepresentation and blue for underrepresentation. FE, pG4s Fold Enrichment; NS, not significant, otherwise *p* < 0.01.

G4Hunter (Bedrat et al., 2016) algorithm was used to identify pG4s within the coding regions in genomic sequences of *A. thaliana*, *O. sativa*, and *H. sapiens*. Supplementary Table S1 shows distributions and frequencies of identified putative pre-mRNA G4s for every tested species. Observed arrangement of pG4s varied greatly between the plant species, with thale cress nearly reaching an order of magnitude lower cumulative distribution of pG4/kbp than rice (Figure 1A). In contrast, rice pG4 density was only slightly lower than that in humans. Based on the genomic location, every predicted pG4 was assigned to corresponding genic structural element: 5′UTR, 3′UTR, intron, CDS, intron/exon junction (InEx), or UTR/CDS junction. To assess possible over- or underrepresentation of pG4s within these features (“Enrichment” column in Supplementary Table S1), we have compared observed pG4 densities with mean values of pG4 density distributions simulated by random shuffling (for details see section Materials and Methods).

The observed increase in the number of pG4s was not uniform along the gene features and varied between the tested species (Figure 1B and Supplementary Table S1). Similar values were detected only within intron/exon and 3′UTR/CDS junctions. In the case of 3′UTR/CDS junctions, in all species, we observed a strong depletion of pG4s. Higher relative distribution of pG4s in CDS regions and 5′UTR/CDS junctions were found uniquely in plants. In all other cases, rice and Arabidopsis differed from each other. Rice and human sequences show very strong overrepresentation of pG4s in 5′UTRs and, to a lesser extent, in introns. In contrast, Arabidopsis introns seem to be depleted of pG4s. Opposite situation can be observed for 3′UTRs, where we observed a high pG4s density in Arabidopsis and humans, but not in rice. All observed enrichment/depletion values, with an exception of 3’UTR/CDS junctions in Arabidopsis, were statistically significant (*p* < 0.01). In general, the distribution profiles were in agreement with the previous studies (Huppert and Balasubramanian, 2005; Mullen et al., 2010; Wang et al., 2015) and confirm the validity of the used annotation methodology.

### Variability of pG4s

**FIGURE 2:**
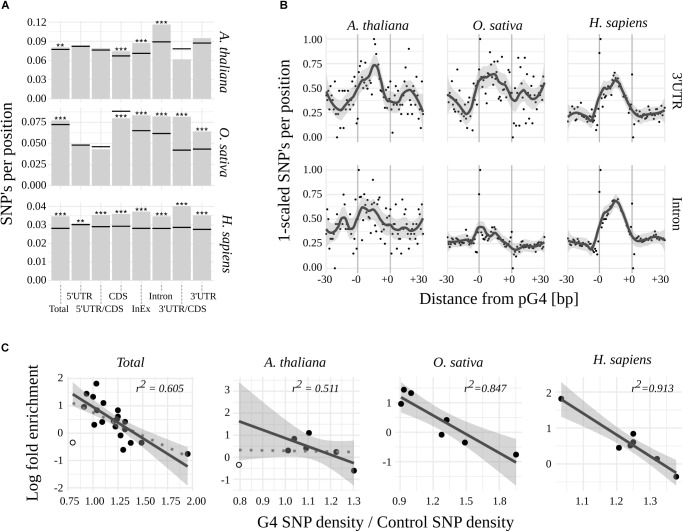
Variability of pG4s. **(A)** SNP densities for pG4s (bars) and their backgrounds (horizontal lines) Stars represent statistical significance at different values of alpha (^∗∗^ 0.01; ^∗∗∗^ 0.001). **(B)** Plots representing SNP density landscape of pG4s and their flanking regions in 3′UTR and Introns. The values were scaled to the range from 0 to 1. pG4s are situated between –0 and +0 marks. **(C)** Relationship between regions’ enrichment with pG4s and proportion between variant density within pgG4s and control variant density. 3′UTR/CDS junction in *Arabidopsis* (hollow circle) was excluded from regression (included in dotted line).

To further evaluate the potential biological significance of G-quadruplexes located within mRNA coding regions, we assessed their sequence variability among 1135 Arabidopsis lines and compared them to publicly available variant data for rice and humans (for details see Materials and Methods). When comparing mean densities of SNPs of pG4s to corresponding gene structural elements (“control”; Figure 2A and Supplementary Table S2), a significantly higher global variability of pG4s could be observed in all three species. The highest sequence dynamics among all tested subjects and gene elements was observed for Arabidopsis in Introns, 3′UTRs (at *p* = 0.055) and Intron/Exon junctions.

In contrast to humans, plants were uniquely characterized by the absence of a significant, relative variability of pG4s in the 5′ part of the mRNAs. Moreover, 5′UTR/CDS pG4s in rice exhibited a hint of apparent invariability (*p* = 0.077). 3′UTR/CDS junction pG4s show high variability in humans and rice, but not in Arabidopsis (where the total count of 3′UTR/CDS pG4s was low at *n* = 10). High sequence dynamics was also observed in the case of CDS pG4s in human and thale cress but interestingly, the opposite relation was found for rice.

We noted that a low variability of pG4s overrepresented in plant 5′UTR/CDS couples with G-quadruplexes’ high variability in underrepresented 3′UTR/CDS in rice and human. This implicated a possibility of reverse correlation between regions’ pG4 enrichment and variability. Indeed, such a correlation appears to exist, most notably in the case of *H. sapiens* and *O. sativa* (Figure 2C). In *A. thaliana* the correlation was not observed, unless low count 3′UTR/CDS pG4s were excluded from the analysis (Figure 2C).

Next, a landscape of distribution of mean SNP densities in the proximity of pG4s (Supplementary Figure S1) has been investigated. The plots obtained for the most variable regions, i.e., 3′UTR and Introns (Figure 2B), clearly show an elevated SNP density within the pG4s in contrast to the 30 bp flanking regions. A similarly sharp distinction can be seen in the case of 5′UTR for all species, as well as CDS for Arabidopsis and humans (Supplementary Figure S1). The pattern was less pronounced, or completely lost, in the case of pG4s within junctions, especially in plants. An additional analysis with the inclusion of Indels using Arabidopsis data further confirmed those observations (Supplementary Figure S2). Interestingly, this analysis revealed a particularly high SNP incidence at the first two positions of pG4s in most cases (Supplementary Figure S1).

**FIGURE 3:**
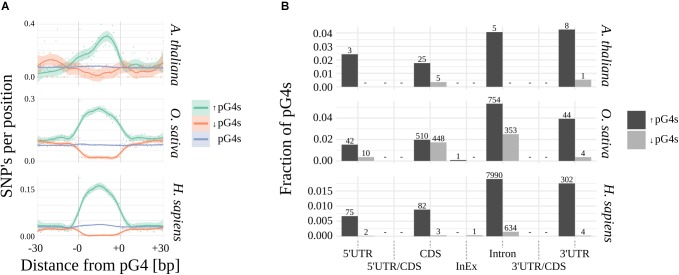
Significantly variable and conserved pG4s. **(A)** Difference in SNP landscapes between significantly variable (green), conserved (red) and general population of pG4s (blue). **(B)** Distribution of significantly variable (black) and significantly invariable (gray) pG4s across gene structural parts. Number of pG4s stated above the bars.

Using Fisher’s exact test, we have identified pG4s that show extreme (lowest and highest) distribution of SNPs compared to their localization regions (Figure 3 and Supplementary Data). The variability landscapes of the extreme fractions show very distinct patterns, consistent with the expectations – i.e., elevation of SNP density in variable pG4s and decrease of SNP density in the conserved pG4s (Figure 3A). In agreement with the results related to general pG4s variability (see above), the highest fraction of significantly variable pG4s was found within 3′UTR’s and Introns, reaching roughly twice the value found for 5′UTRs and CDS (Figure 3B). In comparison, the group of conserved pG4s was notably smaller. 5′UTR and Introns in Arabidopsis represent an extreme example, where no invariable pG4s were found. Interestingly, a particularly high percentage of conserved pG4s has been found in rice, especially in CDS and Introns. In general, none or only few significantly variable or conserved pG4s were found within UTR/CDS and InEx junctions.

### ΔG4Hscore Analysis

In order to elucidate the significance of pG4s variation, we examined how the observed differences affect the assembly potential of the G4 structure. On account of the lack of thermodynamic model of G4 stability, we tested whether the variant substitution generates a shift in the distribution of the ΔG4Hscore (observed change of G4Hscore) in relation to the background distribution (substitution probabilities equal to those of the whole genic region). We have considered two complementary models. First, every variant-containing pG4 was described with a multiset of ΔG4Hscore across all possible single substitutions (for details see Materials and Methods). This analysis revealed a general preference toward variants lowering G4Hscore (Supplementary Figure S3). The second approach was constrained by fixing the variant positions (for details see Materials and Methods). This allowed us to assess the effect of observed substitutions only, regardless of their position in pG4s. In all species and genic features, we observed a slight, yet consistent, skew toward lower G4Hscore values at the higher end of the distributions (Supplementary Figure S4). It has to be noted, however, that it was statistically significant only in high count samples, i.e., UTRs and Introns in humans and CDS in all species. This suggests that the substitutions elevating G4Hscore, and thus increasing the probability of G4 forming, might be a subject to a cleansing selection, however, further validation of this conclusion will be necessary. Interestingly, with fixed positions there is no observable global shift of distribution of ΔG4Hscore. Taken together, the results suggest a preference toward substitutions with a destabilizing influence, which is affected mostly by the position but not by the type of substitution. Overall, the results suggest that observed higher sequence variability of pG4s in various mRNA regions, coupled with propensity of variants toward lowering the G4Hscore may be a result of a positive force toward destabilization of the G-quadruplex structures.

## Discussion

We explored the variability of sequences potentially forming G-quadruplexes in two plant species – *A. thaliana* and *O. sativa* in reference to humans. The analysis was narrowed down to pG4s present in pre-mRNA encoding genomic regions. As the analysis concerned functional distribution of pG4s, we first re-assessed the arrangement propensity of pG4s in studied genomes. As reported previously (Huppert and Balasubramanian, 2005; Mullen et al., 2010; Wang et al., 2015), human and rice pG4 densities were significantly higher than in Arabidopsis. Interestingly, it has been shown that the majority of human and yeast RNA G-quadruplexes are unfolded, possibly due to the involvement of RNA-binding proteins (RBPs) (Guo and Bartel, 2016). Given the high density of pG4s in the human genome, such mechanism seems to be legitimate. Bacteria, on the other hand, use a different strategy – their RNAs are depleted of pG4s (Guo and Bartel, 2016). Because there is a large disproportion in pG4s density between monocots and dicots, it would be interesting to see, whether the global transcript G4 resolvement stands true for any of them.

In general, pG4 distribution patterns varied between the subjects and were not uniform across the structural regions of the genes in any of the cases. The most pronounced differences between the species were observed for UTRs, suggesting a possibility of varying mechanisms of G-quadruplex involvement in the processes taking place at those sites. Intriguingly, the stop codon-containing 3′UTR/CDS pG4s are avoided in all studied species, which might suggest detrimental influence of G4s on translation termination. Observed pG4s’ and profiles of distribution were concordant with previous reports, despite expanding the pool of pG4s with non-canonical sequences. This suggests that non-canonical and canonical pG4s behave similarly in the genomic context, and further supports the necessity of including the former in the future studies.

The analysis of the pG4s variation revealed that in general pre-mRNA pG4s are affected by a greater variability than the surrounding sequences. Our results concerning human pG4s mutability are concordant with that of Du et al. (2013). Earlier studies by Nakken et al. suggested that pG4s are less variable than the background (Nakken et al., 2009). Du et al. (2013) proposed that the differences between their results might have arisen as a consequence of considering only SNP variability and exclusion of repetitive regions. We show that SNP analysis in repeat masked genome, when narrowed down to pre-mRNA, is sufficient to support Du X’s findings, even after expanding and redefining the pool of pG4s.

Although in both plants and humans, the general population of pG4s exhibited a significant variability, their profiles across functional regions differed. Most notably: in both plant species, the variability of 5′ ends’ pG4s was not significantly elevated. In the case of human 5′UTRs, the difference in variability between pG4s and background, although statistically significant, is relatively small. However, the AUG-containing 5′UTR/CDS junctions show significant variability (in contrast to plants), which is especially interesting, given that plant, but not human, genomes are enriched with pG4s in these regions. Concurring with the known mutagenic nature of G4s, one can speculate whether the diminished variability of pG4s in 5′ end may be a result of a counterbalancing mutagenicity of the regions. This would seem to be plausible, considering so far supported regulatory role of 5′ end G4s. Altogether plant 5′UTR/CDS pG4s appear to be an interesting target for further experimental studies.

Another interesting example of differences in the sequence variability of pG4s can be observed in CDS regions. Here, the pG4s in rice are invariable and the highest rate of changes can be observed in humans. pG4s within coding regions have been shown to impair ribosome progression (Endoh and Sugimoto, 2016), and recently have been proposed to act as triggers of novel type of mRNA degradation (Ibrahim et al., 2018). Moreover, CDS are regions of complex selection pressure, where the sequence does not only determine the mRNA structure, but also composition of downstream protein product. Given that pG4s are bound to happen in stretches of G-rich codons, it is therefore interesting to speculate, whether the pG4s enrichment in this region, and – in the case of rice – apparent invariability, is not just a result of an acceptable genetic drift.

Interestingly, we have noticed a hint of inverse correlation between regions’ pG4s enrichment and variability of pG4s. Assuming mutagenicity of G4s to be constant across the regions, this result suggests that the accumulation of pG4s in certain regions might be less tolerable, and their increased observed variability may be an indication of the selection. This hypothesis is further supported by the observation that, in general, substitutions that are more likely to be detrimental to G4s formation seem to be overrepresented. Therefore, it might appear that on a large scale the emergence of G4s is detrimental, and sequence variation serves as a mechanism to prevent their formation. Consequently, the significantly invariable pG4s identified within regions of high pG4 variability might be interesting target for experimental studies. There is also a possibility that G-quadruplexes and other mutagenic non-canonical nucleic acid forms may act as evolutionary drivers – their high mutagenicity coupled with regulatory potential could provide an option to regulate gene expression in evolutionary, rather than physiological, timeframes.

## Author Contributions

PK performed all the calculations, analyzed the results, and wrote the manuscript. WK conceived the project, supervised the calculations, discussed the results, and wrote the manuscript.

## Conflict of Interest Statement

The authors declare that the research was conducted in the absence of any commercial or financial relationships that could be construed as a potential conflict of interest.

## Footnotes

1 http://broadinstitute.github.io/picard/
