# Supplementary figures and images for: Sequence Dynamics of Pre-mRNA G-Quadruplexes in Plants
Source: Front Plant Sci. 2019 Jun 27;10:812. doi: 10.3389/fpls.2019.00812 (PMC6610454; doi:10.3389/fpls.2019.00812)

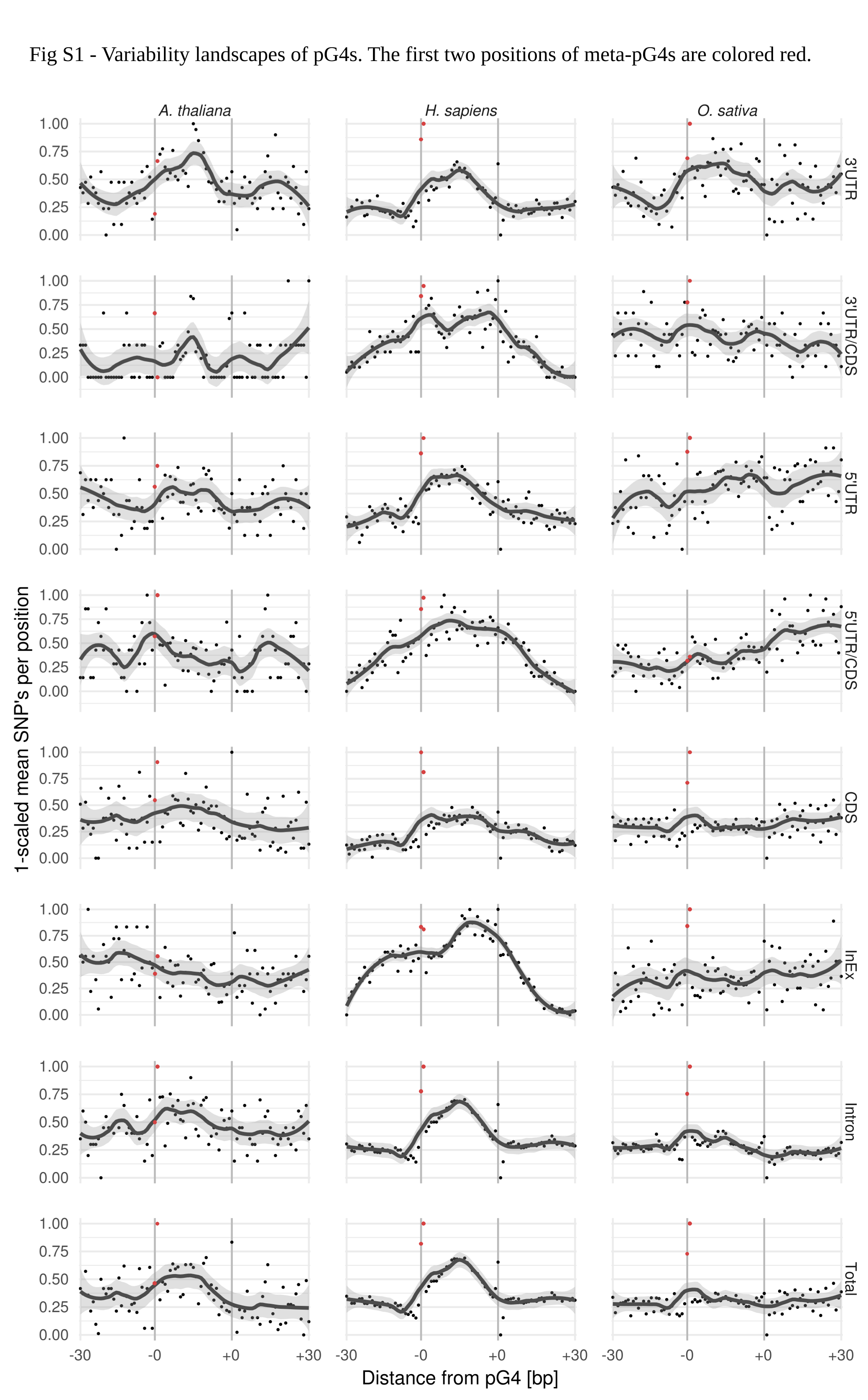

Supplement: Supplementary file 1 [file Image_1.TIFF]

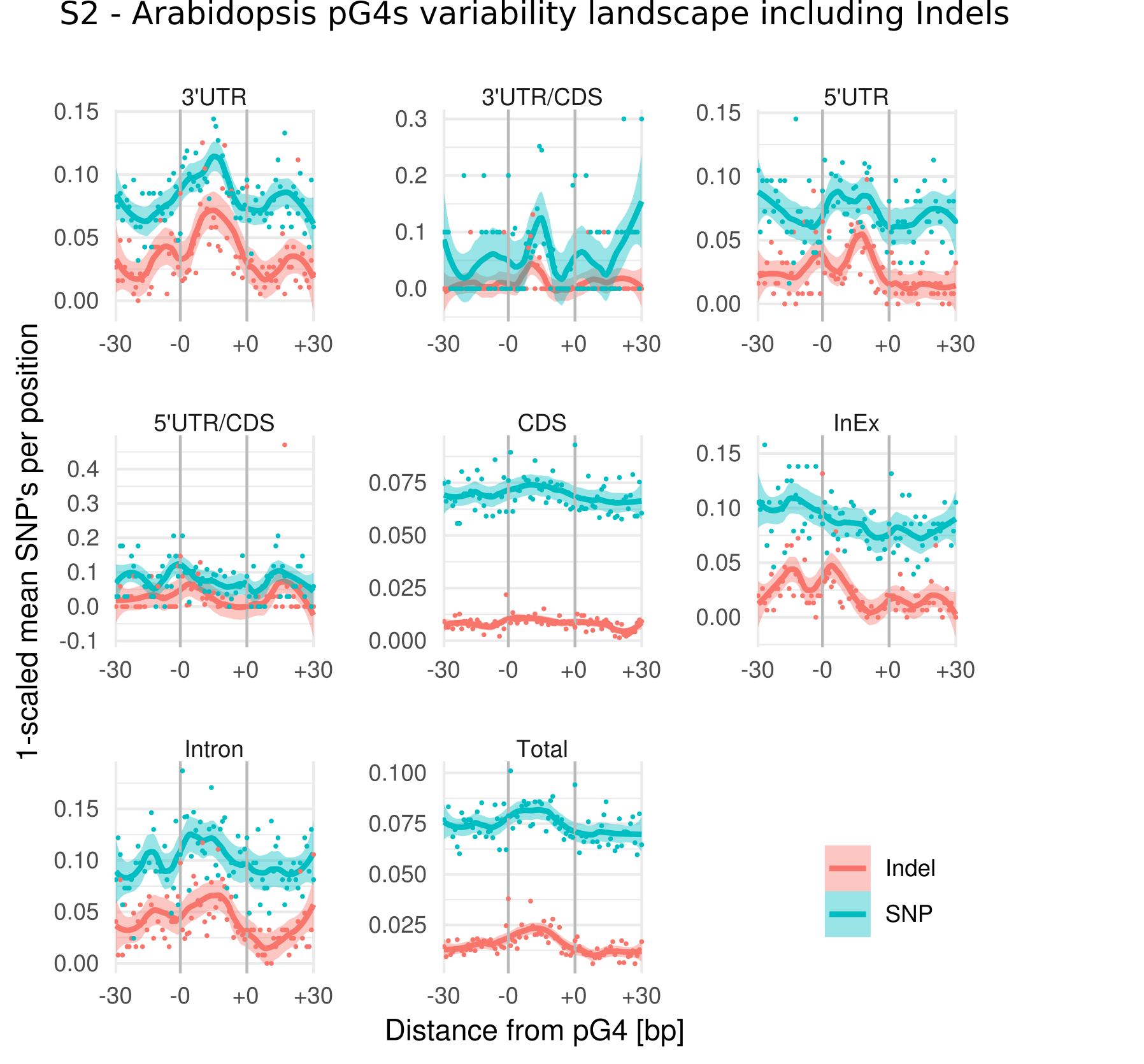

Supplement: Supplementary file 2 [file Image_2.TIFF]

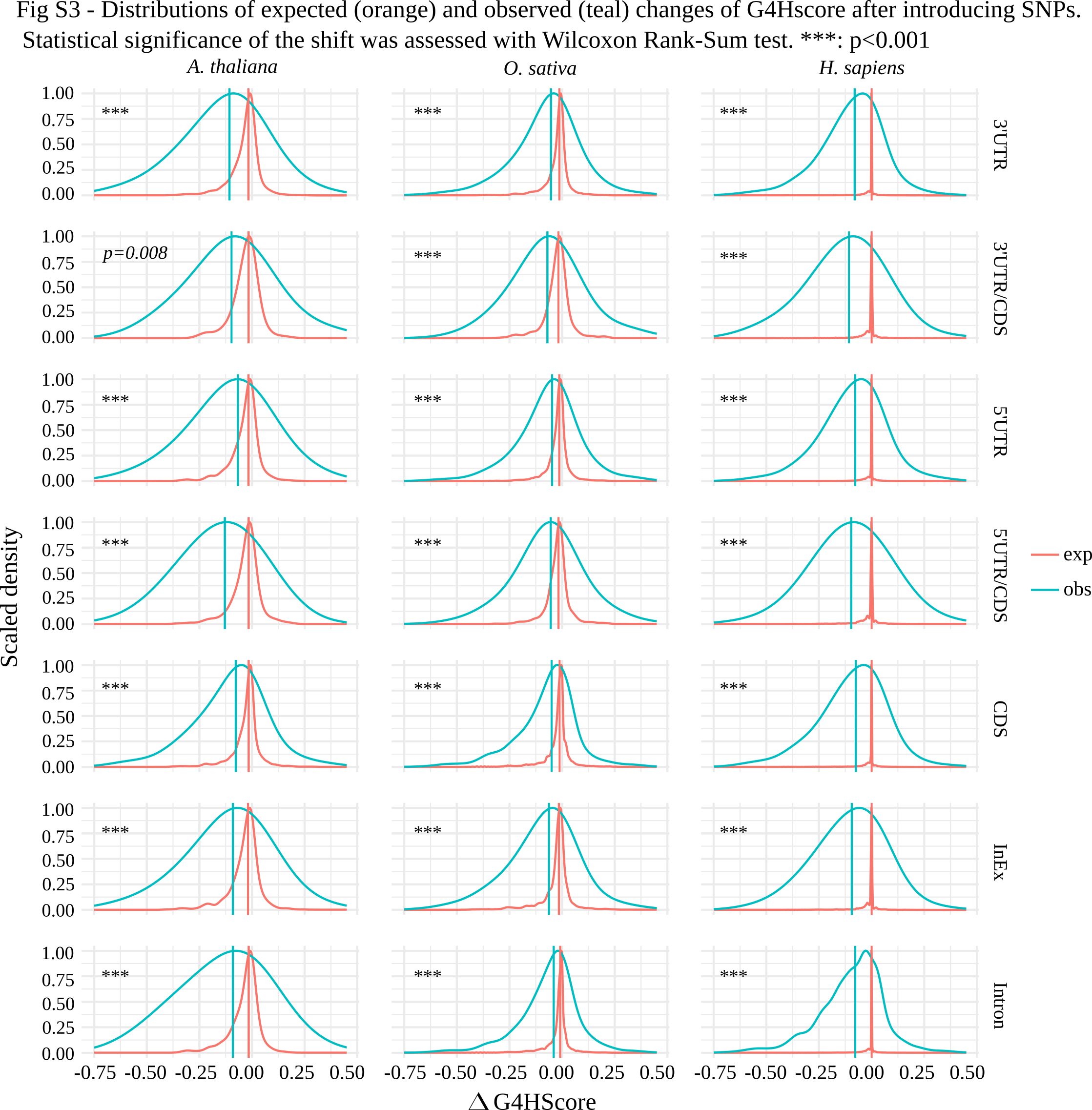

Supplement: Supplementary file 3 [file Image_3.TIFF]

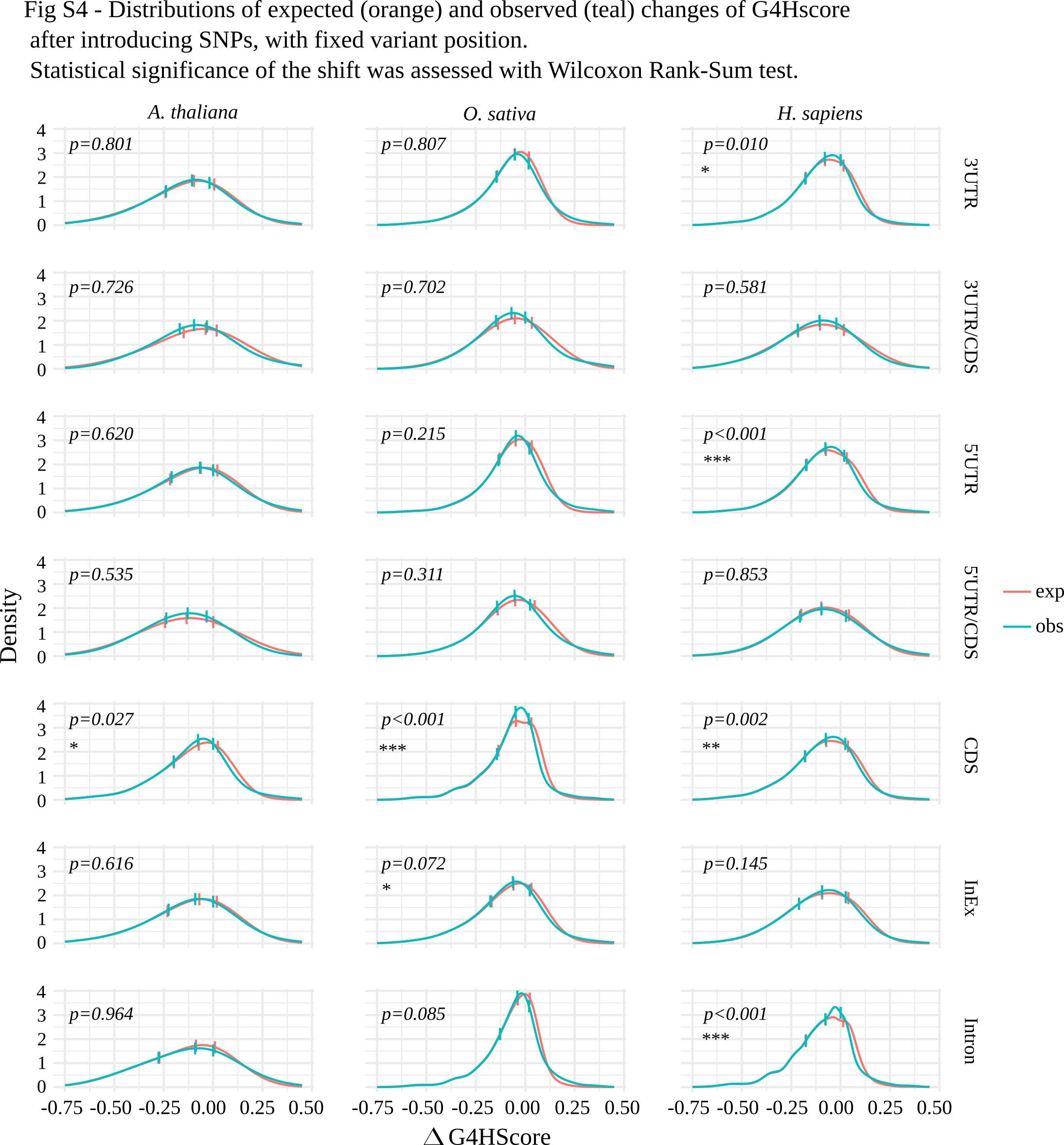

Supplement: Supplementary file 4 [file Image_4.TIFF]
