# Supplementary material for: Sequence Dynamics of Pre-mRNA G-Quadruplexes in Plants
Source: Front Plant Sci. 2019 Jun 27;10:812. doi: 10.3389/fpls.2019.00812 (PMC6610454; doi:10.3389/fpls.2019.00812)
Supplement: Supplementary file 5 [file Data_Sheet_2.PDF]

Table_S1. Distribution of pG4s within coding genetic elements among three tested species.

	Arabidopsis thaliana			*Oryza sativa*			*Homo sapiens*		
	No. of pG4s	pG4s/kbp	Enrichment	No. of pG4s	pG4s/kbp	Enrichment	No. of pG4s	pG4s/kbp	Enrichment
Total	2028	0.031	n/a	47032	0.285	n/a	484383	0.382	n/a
5'UTR	124	0.019	1.359	2756	0.448	3.777	11241	1.045	6.129
5'UTR/CDS	34	0.019	2.313	451	0.243	4.234	492	0.140	1.566
CDS	1419	0.042	3.016	25788	0.334	2.621	9282	0.262	1.668
InEx	152	0.011	1.273	1223	0.057	0.920	2730	0.104	1.151
Intron	123	0.007	0.544	14108	0.196	1.515	421348	0.358	1.832
3'UTR/CDS	10	0.006	0.709^{NS}	51	0.028	0.468	248	0.062	0.701
3'UTR	188	0.024	1.506	1118	0.099	0.705	17255	0.444	2.313

Table_S2. Variability of pG4s.

	Arabidopsis thaliana				*Oryza sativa*				*Homo sapiens*			
	Mean SNP density	Control SNP density	X^2	p	Mean SNP density	Control SNP density	X^2	p	Mean SNP density	Control SNP density	X^2	p
Total	0.080	0.077	7.623	0.006	0.077	0.072	594.044	<0.001	0.035	0.028	25708.8	<0.001
5'UTR	0.083	0.082	0.054	0.816	0.048	0.048	0.055	0.815	0.031	0.030	8.108	0.004
5'UTR/CDS	0.079	0.076	0.119	0.730	0.043	0.046	3.117	0.077	0.035	0.029	20.515	<0.001
CDS	0.074	0.067	31.958	<0.001	0.080	0.088	737.371	<0.001	0.036	0.029	455.500	<0.001
InEx	0.087	0.071	14.732	<0.001	0.083	0.065	185.623	<0.001	0.037	0.028	234.492	<0.001
Intron	0.116	0.089	27.199	<0.001	0.082	0.062	3635.75	<0.001	0.035	0.028	22671.5	<0.001
3'UTR/CDS	0.062	0.078	1.058	0.304	0.082	0.042	57.165	<0.001	0.040	0.029	38.567	<0.001
3'UTR	0.095	0.087	3.678	0.055	0.064	0.043	379.932	<0.001	0.035	0.028	1238.7	<0.001
